# Evaluation of the Fracture Liaison Service within the Canadian Healthcare Setting

**DOI:** 10.1155/2020/6742604

**Published:** 2020-03-21

**Authors:** Matthew Wong-Pack, Nawazish Naqvi, George Ioannidis, Ramy Khalil, Alexandra Papaioannou, Jonathan Adachi, Arthur N. Lau

**Affiliations:** ^1^Department of Medicine, University of Toronto, Toronto, ON, Canada; ^2^Division of Rheumatology, Department of Medicine, Faculty of Health Sciences, McMaster University, Hamilton, ON, Canada; ^3^Geriatric Education and Research in Aging Sciences (GERAS), St Peter's Hospital, Hamilton, ON, Canada

## Abstract

Previous studies evaluating fracture liaison service (FLS) programs have found them to be cost-effective, efficient, and reduce the risk of fracture. However, few studies have evaluated the clinical effectiveness of these programs. We compared the patient populations of those referred for osteoporosis management by FLS to those referred by primary care physicians (PCP), within the Canadian healthcare system in the province of Ontario. Specifically, we investigated if a referral from FLS is similarly effective as PCP at identifying patients at risk for future osteoporotic fractures and if osteoporosis therapies have been previously initiated. A retrospective chart review of patients assessed by a single Ontario rheumatology practice affiliated with FLS between January 1, 2014, and December 31, 2017, was performed identifying two groups: those referred by FLS within Hamilton and those referred by their PCP for osteoporosis management. Fracture risk of each patient was determined using FRAX. A total of 573 patients (*n* = 225 (FLS group) and *n* = 227 (PCP group)) were evaluated. Between the FLS and PCP groups, there were no significant differences in the absolute 10-year risk of a major osteoporotic fracture (15.6% (SD = 10.2) vs 15.3% (SD = 10.3)) and 10-year risk of hip fracture (4.7% (SD = 8.3) vs 4.7% (SD = 6.8)), respectively. 10.7% of patients referred by FLS and 40.5% of patients referred by their PCP were on osteoporosis medication prior to fracture. Our study suggests that referral from FLS is similarly effective as PCP at identifying patients at risk for future osteoporotic fractures, and clinically effective at identifying the care gap with the previous use of targeted osteoporosis therapies from referral from PCP being low and much lower in those referred by FLS. Interventional programs such as FLS can help close the treatment gap by providing appropriate care to patients that were not previously identified to be at risk for fracture by their primary care physician and initiate proper medical management.

## 1. Introduction

Osteoporosis, a skeletal disorder, is characterized by compromised bone strength predisposing individuals to an increased risk of fracture. In particular, over 80% of fractures occurring in patients over the age of 50 can be attributed to osteoporosis [1]. The most common of these osteoporotic fractures include breaks in the vertebrae of the spine, bones of the forearm/wrist, and the hip, with such fractures being a source of substantial morbidity for individuals suffering from osteoporosis [2]. Depending on the fracture type, the 5-year mortality rate postfracture can increase from 1.3 to 13.2 per 100-person years for women, and from 2.7 to 22.3 per 100-person years for men [3]. Leading to serious morbidity, disability and healthcare costs, osteoporosis poses a significant burden on modern economies. Illustrating the growing economic burden of osteoporosis within Canada, the economic cost of osteoporosis is estimated to have grown from $1.3 billion dollars in 1993 to $4.6 billion dollars by 2016 [4]. Despite this growing economic cost and significant morbidity of osteoporosis, less than 20% of patients with fragility fractures are examined or treated with antiresorptive treatment to increase bone strength, demonstrating a concerning care gap for osteoporotic patients [3].

To ensure that this “osteoporosis care gap” is addressed, multidisciplinary care models such as the fracture liaison service (FLS) have been developed and implemented across various countries, including Canada. The model focuses on the (1) identification of patients aged 50 years and older presenting to a hospital with either a new fragility fracture and/or newly reported vertebral fracture, (2) assessment and stratification of the patient's fracture risk, and (3) initiation of the appropriate osteoporosis medications and nonpharmacological interventions [5, 6].

Studies on FLS examining improvements in patient care, reductions in fracture risk, and minimalization of overall healthcare costs have found this service provision to be effective [7, 8]. In Canada, a study examining a coordinator-based FLS at St. Michael's Hospital in Toronto, Ontario, showed that having such a program in place resulted in a high rate of education, evaluation, and pharmacological treatment of patients' postfracture [9]. Furthermore, an additional study performed at this center found this service provision improved quality-adjusted life-years by 4.3 years in patients.

Many studies have provided promising results on FLS' abilities to mitigate costs and improve patient care. This has led to this service provision being implemented across Ontario in high-demand locations including Toronto, Ottawa, London, and Hamilton. However, few studies have evaluated the effectiveness of this service provision from the clinical perspective. However, few compared the patient population of those referred from this program to patients referred from the primary care for osteoporosis management within the Canadian Healthcare system. Thus, the objective of this study was to evaluate the differences in fracture risk and bone health of patients referred by FLS to those referred by their primary care physician within the surrounding area. The second outcome of interest examined the initiation of pharmacotherapy for osteoporosis in each group before and after the osteoporosis assessment. Collectively, these two metrics will be used to determine if there is an underlying care gap that is being addressed by this program.

## 2. Materials and Methods

### 2.1. Study Design

This retrospective cohort study was conducted using electronic medical records (EMR) of patients that were referred to the a single-academic rheumatology practice through the fracture liaison service (FLS) program within Hamilton and those that were referred to the clinic for osteoporotic care from their primary care physician (PCP). All patients referred from FLS, and their PCP were first screened using a search query within the EMR program from 01/01/2014 to 31/12/2017 for osteoporosis management using the operative code of “*fracture*” and “*osteoporosis*,” respectively, for each group.

Eligible patients referred by FLS satisfied the following inclusion criteria: assessed by FLS in Hamilton, Ontario and referred to the Hamilton Rheumatology Medical Practice (HRMP) in Hamilton, Ontario; 18 years of age; and sustained a “fragility fracture (fracture occurring spontaneously or following minor trauma such as a fall from standing height or less).” No patients assessed by FLS were excluded in order to maintain patient heterogeneity. For the patients referred by the PCP for osteoporosis management, given that the number of non-FLS referred patients exceeded the number of patients referred through FLS, a random sample of the PCP-referred patients was obtained. These patients were not required to have sustained a fracture to satisfy the referral criteria, which reflects the current standard of care. These patients were randomly selected using a computerized program from a list of all patients referred for osteoporosis assessment by their PCP between 2011–2017 and were matched 1 : 1 to the number of referred patients through FLS. Study approval was obtained from the Hamilton Integrated Research Ethics Board (REB tracking number 3542-C).

### 2.2. Data Collection

All patients referred by FLS and those by their PCP were assessed by a rheumatologist to determine their risk of subsequent fracture. Bone mineral density (BMD) measurements of the lumbar spine, femoral neck, and hip by dual-energy X-ray absorptiometry (DXA) machine were obtained retrospectively. Fractures were classified into major osteoporotic (hip, spine, wrist, or humerus) and nonmajor osteoporotic fractures [10]. In addition to this information, variables collected included demographic information, family history, comorbidities, medication use, recent BMD scans, and prior fractures.

### 2.3. Statistical Analysis

Using the combined information gathered from the BMD and clinical risk factors, patients' 10-year fracture risks for a major osteoporotic fracture and hip fracture were calculated using the Fracture Risk Assessment Tool (FRAX) algorithm utilizing reference values derived from a Canadian population. Descriptive statistics are presented as means (standard deviation) for continuous and counts (percentage) for categorical variables for the FLS and PCP groups respectively. Differences between the groups were calculated using MedCalc Statistical Software Version 14.12.0 (MedCalc Statistical Software BVBA, Ostend, Belgium), and descriptive statistics were summarized using SAS version 9.3 for Windows (SAS Institute Inc., Cary, NC, USA). For continuous variables, two-sample *t*-tests were used to determine differences between groups. For categorical variables, Chi-squared tests were used to determine differences between groups. Statistical significance was set at 0.05.

## 3. Results

**Figure 1:**
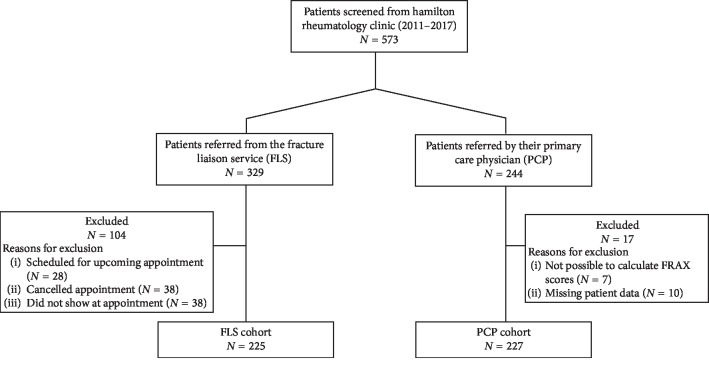
Enrollment of patients.

We identified 329 patients who were assessed by FLS between 2014 and 2017. Those who were scheduled for an upcoming appointment, cancelled their appointment, or did not attend their appointment were excluded. Additionally, patients for which it was not possible to calculate FRAX scores for or had missing patient data were excluded. Two hundred and twenty-five patients were included in the FLS cohort and were matched 1 : 1 with a random selection of 227 patients referred by their PCP within the same time period (Figure 1).

Baseline demographics are provided in Table 1. There were no significant differences in the gender, age, body mass index, menopausal status, smoking history, and alcohol use between patients referred by FLS and patients referred by their PCP. Comorbidities, risk factors, and medications are provided in Table 2. Compared with the patients referred by their PCP, patients referred by FLS had a higher frequency of diabetes, and cardiovascular disease. However, patients referred by their PCP had a higher frequency of chronic renal failure and endocrine disorders compared to those referred by FLS. More patients referred by their PCP consumed Vitamin D and higher dosages of calcium per day. There were no significant differences in proton pump inhibitor use between groups. The frequency of selective serotonin reuptake inhibitor/tricyclic antidepressant use was higher in the FLS group, while glucocorticoid usage was greater in patients referred by the PCP.

BMD, fracture history, and fracture risk are provided in Table 3. Patients referred by FLS had greater BMD in the lumbar spine (0.952 (SD = 0.175) g/cm^2^ vs 0.881 (SD = 0.170) g/cm^2^), femoral neck (0.687 (SD = 0.140) g/cm^2^ vs 0.646 (SD = 0.115) g/cm^2^), and total hip (0.822 (SD = 0.160) vs 0.746 (SD = 0.117) g/cm^2^) compared to those referred by their PCP. 223 (99.1%) patients referred by FLS presented with a fracture compared to 45 (19.8%) patients referred by their PCP. The majority of patients referred by FLS sustained fractures of the elbow, shoulder, and spine while patients referred by their PCP sustained fractures of the wrist.

There were no significant differences in between patients referred by FLS compared to those referred by their PCP with respect to their absolute 10-year risk of a major osteoporotic fracture (15.6% (SD = 10.2) vs 15.3% (SD = 10.3)) and 10-year risk of hip fracture (4.7% (SD = 8.3) vs 4.7% (SD = 6.8)), respectively. Only 10.7% of patients are referred by FLS, while 40.5% of patients referred by their PCP were on osteoporosis medication prior to fracture despite having similar fracture risks. Upon assessment, the majority of patients in both the FLS and PCP groups (60.9% and 72.7%) were either initiated on therapy or had their osteoporosis medication changed and those for which fracture risk was deemed low were initiated on a combined regimen of vitamin D and calcium supplementation.

## 4. Discussion

Our data from this study showed that the patients who are referred by FLS and those referred by PCPs had no significant difference in the 10-year probabilities of a major osteoporotic (15.6% vs 15.3%) or hip fracture (4.6% vs 4.6%) between groups as determined by the FRAX algorithm. This supports that programs such as FLS are needed in order to identify, investigate, and initiate treatment in patients that would have otherwise gone undiagnosed and been at a risk for future, subsequent fractures. These are patients whose bone densities are higher despite having sustained a fracture.

A cross-sectional study conducted among men and women with a recent clinical vertebral and nonvertebral fracture who were evaluated at an FLS in the Netherlands found that comorbidities and medications associated with an increased bone- or fall-related fracture were present in two-thirds of patients attending a FLS after a recent fracture [11]. Comorbidities were classified using the ICD-10 classification and medications according to the anatomic therapeutic chemical (ATC) classification. While we did not formally classify our comorbidities or medication usage, the results of Vranken et al. are similar to our findings in that patients referred by the FLS and by their primary care physician present with several comorbidities associated with fracture risks at assessment.

Although the mean bone mineral densities and *t*-scores were higher in patients referred by the FLS compared to those by their primary care physician (*p* < 0.001), this was not the sole determining factor for whether a patient was initiated on osteoporosis medication. It is important to recognize that patients at a high risk of fracture that do not necessarily have *T* scores of less than −2.5 can have osteopenia (*T* score of −1.0 to −2.5) in combination with other comorbidities, as shown in our study. Treatment decisions were determined by evidence-based practice methods such as utilizing FRAX national osteoporosis guidelines.

Several authors have attempted to classify and group FLS into specific models of care. Within each model, there was a common element of having an individual, such as a clinical nurse, who coordinates care for patients to specialist physicians based on specific protocols [12, 13]. Ganda et al. described that models that are less effective often either (1) identify and investigate patients but then refer care back to their primary care physician for initiation of treatment, (2) identify patients at risk and inform them and their primary care physician but do not undertake any assessment or treatment of patients, or (3) identify at-risk patients and inform them and educate them but take no further part in communicating their findings to other stakeholders in the patient's care. FLS models that identify, investigate, and initiate treatment have shown to be the most effective at fracture prevention. Both populations within our study follow this approach by having patients assessed by a specialist for further evaluation and treatment. Programs such as FLS with interventions are needed to ensure that those who are not referred by their primary care physician receive appropriate care to decrease the risk for subsequent fracture.

Several strengths of our study include the availability of detailed clinical information, which provides real-world applicability of the FLS in the clinical setting. A significant number of studies have focused on issues pertaining to the implementation of such programs and the processes associated with their development. However, fewer studies have investigated the real-world effectiveness of the program itself from a clinical perspective. This is one of the few studies that directly compare the fracture risk of patients referred by FLS compared to those referred by their primary care physician using FRAX.

The results of our study should be interpreted with the consideration that the study sample is limited to patient referrals by FLS to a single-academic rheumatology practice. Additionally, due to the retrospective nature of the study, data are highly dependent on source records.

## 5. Conclusion

In conclusion, the similarities in FRAX scores between those assessed by the FLS and those referred by primary care physicians suggest that the FLS is effective at targeting patients at risk for future osteoporotic fractures. Additionally, the significant differences between groups in osteoporotic medication use prior to assessment despite having similar fracture risk scores highlights a gap in fracture prevention care within the general population. Programs such as FLS serve to address this gap by providing medical care to previously unrecognized patients at risk for fracture and prevent subsequent future fractures.

## Figures and Tables

**Table 1:** Participant characteristics.

Baseline demographics	FLS group	PCP group	*P* value
Women, *n* (%)	176 (78.2)	192 (84.6)	0.08
Age, years (SD)	66.7 (11.6)	65.8 (11.4)	0.39
Body mass index, kg/m^2^ (SD)	28.9 (6.1)	28.2 (36.0)	0.78
Postmenopausal status, *n* (%) (female)	167 (94.9)	182 (94.8)	0.93
Smoking status, *n* (%)			0.11
Past	11 (4.9)	4 (1.8)	
Current	39 (17.3)	33 (14.5)	
Never	175 (77.8)	190 (83.7)	
Alcohol 3 or more units/day, *n* (%)	22 (9.8)	31 (13.7)	0.20

**Table 2:** Comorbidities, risk factors, and medications.

Comorbidities	FLS group	PCP group	*P* value
Diabetes, *n* (%)	34 (15.1)	18 (7.9)	0.02
Lung disease, *n* (%)	27 (12.0)	31 (13.7)	0.60
Liver disease, *n* (%)	4 (1.8)	5 (2.2)	0.75
Chronic renal failure, *n* (%)	13 (5.8)	38 (16.7)	<0.001
Cardiovascular disease, *n* (%)	111 (49.3)	86 (37.9)	0.01
Endocrine disorder, *n* (%)	17 (7.6)	40 (17.6)	0.001
Inflammatory bowel disease, *n* (%)	5 (2.2)	11 (4.9)	0.13
Celiac disease, *n* (%)	1 (0.4)	5 (2.2)	0.10
Rheumatoid arthritis, *n* (%)	1 (0.4)	4 (1.8)	0.18
Family history of cancer, *n* (%)	107 (48.0)	107 (47.1)	0.86
Cancer/radiotherapy treatment, *n* (%)	27 (12.0)	35 (15.4)	0.29
Vitamin D consumption, *n* (%)	148 (65.8)	197 (86.8)	<0.001
Mean (SD) dosage, IU/day	2180.4 (5673.4)	1837.7 (1447.8)	0.42
Calcium consumption, *n* (%) (dietary and supp.)	215 (95.6)	217 (96.4)	0.63
Dietary calcium consumption, *n* (%)	533.7 (350.1)	612.4 (382.0)	0.03
Supplemental calcium consumption, *n* (%)	62 (27.6)	66 (29.1)	0.72
Mean (SD) dosage, IU/day	665.9 (394.9)	822.0 (366.5)	0.02
PPI use, *n* (%)	60 (26.7)	53 (23.4)	0.42
SSRI/TCA use, *n* (%)	48 (21.3)	25 (11.0)	0.003
Glucocorticoid usage, *n* (%)	6 (2.7)	15 (6.6)	0.04

**Table 3:** Bone mineral density, fracture history, and fracture risk.

Participant characteristics	FLS group	PCP group	*P* value
Mean (SD) BMD lumbar spine (g/cm^2^)	0.952 (0.175)	0.881 (0.170)	<0.001
Mean (SD) BMD femoral neck (g/cm^2^)	0.687 (0.140)	0.646 (0.115)	<0.001
Mean (SD) BMD total hip (g/cm^2^)	0.822 (0.160)	0.746 (0.117)	<0.001
Mean (SD) *T*-score lumbar spine	−0.991 (1.551)	−1.70 (1.470)	<0.001
Mean (SD) *T*-score femoral neck	−1.580 (1.145)	−2.111 (0.884)	<0.001
Mean (SD) *T*-score total hip	−1.112 (1.21)	−1.780 (0.900)	<0.001
Family history of osteoporosis, *n* (%)	66 (29.7)	91 (40.1)	0.02
Family history of hip fracture, *n* (%)	42 (18.9)	38 (16.7)	0.55
Fracture at assessment, *n* (%)	223 (99.1)	45 (19.8)	<0.001
Nature of fracture, *n* (%)			<0.001
Ankle	7 (3.1)	2 (0.9)	
Clavicle	4 (1.8)	0 (0.0)	
Elbow	24 (10.7)	1 (0.4)	
Femur	2 (0.9)	0 (0.0)	
Foot	1 (0.4)	5 (1.1)	
Forearm	4 (1.8)	0 (0.0)	
Hip	12 (5.3)	5 (2.2)	
Humerus	21 (9.3)	0 (0.0)	
Leg	11 (4.9)	2 (0.9)	
Pelvis	3 (1.3)	1 (0.4)	
Shoulder	23 (10.2)	0 (0.0)	
Spine	22 (9.8)	19 (8.4)	
Wrist	89 (39.6)	5 (2.2)	
Other	2 (0.9)	5 (2.2)	
None	0 (0.0)	182 (80.2)	
Fall at assessment, *n* (%)	206 (91.6)	37 (16.3)	<0.001
On prior bone medication therapy, *n* (%)	24 (10.7)	92 (40.5)	<0.001
Bone medication initiated *n* (%)	137 (60.9)	165 (72.7)	0.01
History of a vertebral fracture, *n* (%)	28 (12.4)	28 (12.3)	0.97
History of a hip fracture, *n* (%)	3 (1.3)	9 (4.0)	0.08
History of a wrist fracture, *n* (%)	34 (15.1)	30 (13.2)	0.56
History of a shoulder fracture, *n* (%)	11 (4.9)	8 (3.5)	0.47
History of major osteoporotic fracture, *n* (%)	79 (35.1)	68 (29.9)	0.24
History of other fractures (nonmajor osteoporotic fractures), *n* (%)	71 (31.6)	5 (2.2)	<0.001
Mean (SD) major osteoporotic fractures	1.3 (0.9)	0.5 (0.8)	<0.001
Mean (SD) other fractures	0.7 (1.0)	0.6 (1.0)	0.25
Mean (SD) total lifetime fractures	2.0 (1.2)	1.1 (1.4)	<0.001
FRAX score (major osteoporotic fracture)	**15.6 (10.2)**	**15.3 (10.3)**	**0.72**
FRAX score (hip fracture)	**4.7 (8.3)**	**4.7 (6.8)**	**1.00**

For categorical variables: Chi-squared test was used to determine differences between groups. For continuous outcomes: two-sample *t*-test was used to determine differences between groups.

## Data Availability

The data used to support the findings of this study are restricted by the Hamilton Integrated Research Ethics Board in order to protect patient privacy. Data are available from Arthur N. Lau (arthur.lau@medportal.ca) for researchers who meet the criteria for access to confidential data.
